# Primary skeletal muscle myoblasts from chronic heart failure patients exhibit loss of anti-inflammatory and proliferative activity

**DOI:** 10.1186/s12872-016-0278-3

**Published:** 2016-05-26

**Authors:** Tahnee Sente, An M. Van Berendoncks, An I. Jonckheere, Richard J. Rodenburg, Patrick Lauwers, Viviane Van Hoof, An Wouters, Filip Lardon, Vicky Y. Hoymans, Christiaan J. Vrints

**Affiliations:** Laboratory of Cellular and Molecular Cardiology, Antwerp University Hospital, Edegem, Belgium; Department of Translational Pathophysiological Research, Cardiovascular Diseases, University of Antwerp, Wilrijk, Belgium; Department of Cardiology, Antwerp University Hospital, Edegem, Belgium; Department of Pediatric Neurology, UZ Brussel, Vrije Universiteit Brussel (VUB), Brussels, Belgium; Department of Pediatrics, Nijmegen Center for Mitochondrial Disorders, Translational Metabolic Laboratory, Radboud University Medical Center, Nijmegen, The Netherlands; Department of Thoracic and Vascular Surgery, Antwerp University Hospital, Edegem, Belgium; Department of Biochemistry, Antwerp University Hospital, Edegem, Belgium; Center for Oncological Research (CORE) Antwerp, University of Antwerp, Wilrijk, Belgium

**Keywords:** Chronic heart failure, Muscle wasting, Myoblast cultures, xCELLigence

## Abstract

**Background:**

Peripheral skeletal muscle wasting is a common finding with adverse effects in chronic heart failure (HF). Whereas its clinical relevance is beyond doubt, the underlying pathophysiological mechanisms are not yet fully elucidated. We aimed to introduce and characterize the primary culture of skeletal muscle cells from individual HF patients as a supportive model to study this muscle loss.

**Methods and results:**

Primary myoblast and myotubes cultures were successfully propagated from the *m. vastus lateralis* of 6 HF patients with reduced ejection fraction (HFrEF; LVEF <45 %) and 6 age and gender-matched healthy donors. HFrEF cultures were not different from healthy donors in terms of morphology, such as myoblast size, shape and actin microfilament. Differentiation and fusion indexes were identical between groups. Myoblast proliferation in logarithmic growth phase, however, was attenuated in the HFrEF group (*p* = 0.032). In addition, HFrEF myoblasts are characterized by a reduced TNFR2 expression and IL-6 secretion (*p* = 0.017 and *p* = 0.016; respectively).

**Conclusion:**

Biopsy derived primary skeletal muscle myoblasts of HFrEF patients produce similar morphological and myogenic differentiation responses as myoblasts of healthy donors, though demonstrate loss of anti-inflammatory and proliferative activity.

**Electronic supplementary material:**

The online version of this article (doi:10.1186/s12872-016-0278-3) contains supplementary material, which is available to authorized users.

## Background

Skeletal muscle wasting is observed in a variety of chronic diseases including chronic heart failure (HF) [1–4]. Muscle wasting is present in approximately 70 % of chronic HF patients [5–7]. The loss of muscle mass during HF has a significant impact on the patients’ quality of life and is associated with high morbidity [6]. The mechanisms that underlie HF-related skeletal muscle wasting, however, are currently not clear. A number of hypotheses have been put forth to explain the loss of muscle mass, some of which are physiologic, including prolonged immobilization and malnutrition, or pathologic, such as insulin resistance, impaired myogenesis and inflammation. Until now, mechanistic data generated in this domain have been confined to animal experiments, human muscle biopsy specimens, C_2_C_12_ mouse myoblasts and L6 myotubes cell lines. Although animal models and immortalized cell lines are convenient sources to study the basic features of muscle cells, they are devoid of typical human traits. Specimens of skeletal muscle tissue have been used mainly to study changes in histologic and (ultra)structural features. In recent years, however, the primary cell culture model has gained wide acceptance [8–10]. Studies indicated that human primary skeletal muscle cells adequately retain phenotypic and genotypic traits of the donor, including morphological, metabolic and biochemical similarities, in a controlled in vitro environment and hence are a highly relevant means to study skeletal muscle alterations in vitro [11–13]. Therefore, by generating in vitro cultures of primary skeletal muscle myoblasts and myotubes from HF patients, we may increase the near future ability of identifying novel mechanisms contributing to loss of skeletal muscle mass in this patient population. In the present study, we aimed to characterize primary cultures of skeletal muscle from HF origin in relation to healthy donor cells.

## Methods

### Patient population and controls

The study population consisted of eight patients with systolic HF as a result of dilated cardiomyopathy or ischemic heart disease, recruited from the Heart Failure Clinic of the Antwerp University Hospital (Edegem, Belgium). All patients had a left ventricular ejection fraction (LVEF) of <45 % (HF with reduced EF; HFrEF) and were classified as New York Heart Association (NYHA) functional class II-III. Patients were on a stable dose of HFrEF medication for at least 1 month prior to enrollment. Exclusion criteria were recent acute coronary syndrome (≤3 months), valvular disease requiring surgery and acute myocarditis or pericarditis. Patients with acute or chronic infections, allergies, cancer, inflammatory diseases, diabetes mellitus treated with thiazolidinediones or fibrates, renal failure and musculoskeletal abnormalities were excluded to avoid possible metabolic interference. Eight subjects matched for age and gender, no medication intake and without any significant medical history were recruited as controls. The study conforms with the principles outlined in the Declaration of Helsinki and was approved by the local Ethics Committee of the Antwerp University Hospital (committee for medical ethics UZA - UAntwerp). All participants provided written informed consent before enrollment.

### Myoblast cell culture

Biopsy samples were collected of the *musculus vastus lateralis* using the Bergstrom needle technique [14]. Muscle specimens were trimmed of connective tissue and fat, minced into ±1 mm^3^ fragments and enzymatically dissociated by a series of incubations in 0.05 % trypsin/EDTA/collagenase. The supernatant of successive dissociations was centrifuged at 230 x g for 5 min. The resulting cell suspension was purified from fibroblasts by a pre-plating step in collagen-coated 25 cm^2^ culture flasks containing skeletal muscle growth medium (SKGM; Lonza, Allendale, NJ) [15]. After 45 min of incubation, the non-adhering cells were transferred into a new collagen-coated flask. Cells were cultured in a humidified 5 % CO_2_ atmosphere at 37 °C and growth medium was refreshed every 2 days until cells reached 70 % confluence. Cultures were tested for myogenicity by immunostaining using the muscle-specific antibody desmin (1:100, D1033, Sigma-Aldrich, St. Louis, MO, USA). Only cultures with a myogenic purity >90 % were used for further analysis. All experiments were analyzed blinded and performed at a similar passage 4.

### Assessment of myotubes differentiation and fusion index

In order to induce myogenic differentiation of myoblasts, growth medium was replaced by differentiation medium consisting of serum free Dulbecco’s Modified Eagle Medium (DMEM)/Ham’s F-12 (1:1; Lonza) supplemented with 2 % (v/v) horse serum (Gibco, Life Technologies, Gent, Belgium). At day 6, cells were fixed in 4 % paraformaldehyde. Myotubes and nuclei were visualized by immunofluorescence using an antibody against desmin and 4′,6-diamidino-2-phenylindole (DAPI), respectively (see “in vitro immunocytochemistry section” for details). Myotubes were defined as cells containing at least three nuclei within one continuous cell membrane [16]. The differentiation index (DI) was calculated as the percentage of desmin-positive cells in relation to the total number of nuclei. In order to quantitatively assess the extent of myoblast fusion, a myogenic fusion index (MFI) was determined as the average number of myogenic nuclei present in multinucleated myotubes. The fusion index was analyzed in duplicate by counting at least 250 nuclei from 10 randomly selected fields of view. The deformed myotubes index (DMI) was evaluated as indicated by Yip et al. and quantified analogously to the MFI [17].

### Proliferation kinetics

Myoblast proliferation and viability were continuously monitored using the xCELLigence Real-Time Cellular Analysis (RTCA) system (Westburg, Leusden, The Netherlands), according to the manufacturer’s guidelines. Briefly, myoblasts were grown in special modified 16-well plates (E-plates) with microelectrodes on the bottom of each well detecting electrical impedance-based attachment, spreading and proliferation of the myoblast cultures. Background impedance (SKGM medium alone) was measured and cells were seeded at a density of 10.000 cells/well in growth medium. After 30 min at room temperature (RT) to allow cell attachment, plates were locked in the RTCA device and electrical impedance was measured and expressed as a dimensionless parameter termed cell index (CI), a relative change in electrical impedance representing cell status [18]. The CI was continuously monitored in 15 min intervals with a programmed signal detection schedule for a total time of 172 h. Two replicates of each culture were run.

### In vitro immunocytochemistry and immunohistochemistry

Cultures were grown on collagen-coated glass chamber slides at a density of 5x10^3^ cells and allowed to adhere for 24 h. Next, cells were fixed in 4 % paraformaldehyde in PBS-D for 20 min at 4 °C and permeabilized in 0.1 % Triton X-100 in PBS-D for 5 min. Cells were incubated with primary antibody overnight and with secondary antibody for 1 h. The following primary mouse antibodies were used: monoclonal anti-desmin (1:200; Sigma-Aldrich), monoclonal anti-α-actinin (1:100; Sigma-Aldrich) and monoclonal anti-α-tubulin (1:200; Sigma-Aldrich), and combined with either a donkey anti-mouse IgG secondary antibody, Alexa Fluor 546 conjugated (1:800; Molecular probes, Eugene, OR, USA) or a goat anti-mouse IgG secondary antibody, Alexa Fluor 546 conjugated (1:800; Vector Laboratories, Burlingame, CA, USA). Actin microfilaments were visualized by applying fluorescein isothiocyanate (FITC)-conjugated phalloidin (50 μg/mL in methanol; Sigma-Aldrich) for 20 min in the dark. Immunohistochemical detection of the senescence marker acetyl-p53 in muscle biopsies and cell cultures was performed using the primary antibody anti-p53 (Acetyl-Lys317; 1:200; Abcam). Nuclei were counterstained with DAPI. Cells were visualized using an EVOS fluorescent microscope (Westburg, Leusden, The Netherlands). From each sample, fifteen microphotographs were captured and analyzed manually. The ultrastructural morphology was analyzed using the software program Image J (Version 1.45 s, National Institutes of Health, USA). All specimens were evaluated by two blinded investigators who were unaware of clinical data and group assignment.

### Flow cytometric analysis

Cell cultures were evaluated for muscle specific marker expression using flow cytometry at several time points during myogenesis. Cells were trypsinized and centrifuged at 230 x g for 5 min. The cell pellet was resuspended in permeabilization buffer for 10 min at a concentration of 1x10^5^ cells/ml and incubated with primary antibodies in the dark for 30 min. The following primary antibodies were used: phycoerythrin conjugated mouse anti-human Pax3 (R&D Systems, Minneapolis, MN, USA), phycoerythrin conjugated rabbit anti-human Pax7 (Bioss Inc., Woburn, MA, USA), phycoerythrin conjugated rabbit anti-human MyoD1 (Bioss Inc.), Alexa fluor 488 conjugated mouse anti-human myogenin (R&D Systems) and rabbit phycoerythrin conjugated anti-human myf6 (MRF4) (Bioss Inc.). Gating was implemented based on negative control staining by using mouse IgG1 Alexa 488 conjugated and mouse IgG2A (R&D Systems) phycoerythrin conjugated isotype antibodies (Bioss Inc.). Cell viability was assessed using the dead cell discriminator dye 7-aminoactinomycin (7-AAD) and Annexin V-FITC (Becton Dickinson, Biosciences, Erembodegem, Belgium). Cells were stained with DRAQ5 (BioStatus Limited; Leicestershire, UK) to exclude cellular debris and non-nucleated cells. Cells were analyzed on a FacsCantoTM II flow cytometer (Becton Dickinson). A minimum of 30.000 events was recorded for each analysis. Data analysis was done with FacsDiva 6.1.2 software. Representative graphs of the muscle specific marker expressions are provided in Additional file 1: Figure S1.

### Senescence-associated β-galactosidase activity

Senescence-associated beta-galactosidase (SA-β-gal) activity was assessed with the *β*-Galactosidase Staining Kit (Biovision Research Products, Palo Alto, CA, USA). The protocol was performed according to the manufacturer’s instructions. In brief, cells were fixed for 10 min at RT and incubated overnight in freshly prepared acidic *β*-gal staining solution containing 5-bromo-4-chloro-3-indolyl *β*-D-galactopyranoside (X-Gal) at 37 °C. Senescence was determined by phase contrast (Olympus Optical Co., Tokyo, Japan) under bright field illumination in 10 randomly selected fields as the ratio of SA-*β*-gal positive (blue) cells to the total number of cells. Analyses were performed in triplicate by two independent observers.

### Cytokine production

Concentrations of tumor necrosis factor-α (TNF-α), interleukin (IL)-6, IL-10, interferon-γ (IFN-γ), and IL-1β were determined in cell culture supernatant by a multiplex enzyme-linked immunosorbent assay (ELISA) technique based on electrochemiluminescence (Meso Scale Discovery (MSD) technology, Meso Scale Diagnostics, Rockville, MD, USA) and according to the manufacturer’s instructions. Plates were read on a SECTOR® Imager 6000 instrument. Data was analyzed using the Discovery Workbench 3.0 software. All standards and samples were measured in duplicate.

### RNA isolation and quantitative real-time polymerase chain reaction (RT-PCR)

Total RNA was extracted using the Qiazol reagent technique followed by RNA cleanup (RNeasy Mini Kit, Qiagen). 1 μg of isolated total RNA was reverse-transcribed using the iScript™ cDNA Synthesis Kit (Bio-rad Laboratories, Nazareth, Belgium). RT-PCR gene-specific forward and reverse primers (Eurofins MWG Operon; Ebersberg, Germany) were designed: TNFR1-F: ‘ACC AGG CCG TGA TCT CTA TG’, TNFR1-R:’CAG CTA TGG CCT CTC ACT CC’, TNFR2-F:’CTC AGG AGC ATG GGG ATA AA”, TNFR2-R:’AGC CAG CCA GTC TGA CAT CT’. PCR amplification with EVAGreen supermix was performed on a CFX96TM Real-Time PCR Detection system (Bio-rad). Gene expression was normalized using the reference genes TATA box binding protein (TBP) and beta-2-microglobulin (B2M). Relative quantification of gene expression levels was performed by analyzing the RT-PCR data using the delta delta Ct (2^-ΔΔCt^) calculation. All samples were run in duplicate.

### Biochemical analyses

Fasted peripheral venous serum was collected from all HFrEF patients and healthy controls. Creatinine, total cholesterol, triglycerides, low-density lipoprotein (LDL) and high-density lipoprotein (HDL) cholesterol levels, glucose and high sensitivity C-reactive protein (hsCRP) were assessed immediately on Dimension Vista 1500 instrumenten (Siemens Healthcare Diagnostics NV/SA, Beersel (Huizingen) Belgium) using reagents from Ortho Clinical Diagnostics. Bioelectrical impedance analysis was used for assessment of body composition (Omron body fat monitor BF 300).

### Statistical analysis

Experimental triplicates or duplicates were averaged for statistical analysis. Categorical variables were compared with the Pearson’s Chi-square (X_2_) test. Mann-Whitney U test was used to compare differences between both groups (HFrEF versus Control). Results are presented as mean ± standard error of the mean (SEM). Differences are considered statistically significant if the *p*-value is less than 0.05 (*P* < 0.05). Statistical analyses were performed using SPSS software (IBM SPSS Statistics Inc, Version 20.0, Chicago, IL, USA).

## Results

### Patient characteristics

The clinical characteristics of the patients and the healthy donors are provided in Table 1. All HFrEF patients received standard HF treatment: 75 % of the patients received a beta-blocker, 62.5 % an angiotensin converting enzyme (ACE)-inhibitor, 87.5 % were on diuretics, 50 % were treated with an angiotensin II receptor antagonist and 75 % were on statin therapy. Age, gender and body mass index (BMI) were similar between groups. HFrEF patients had a pro-inflammatory blood profile (hsCRP, *p* = 0.007) and a dyslipidemic state (triglycerides, *p* = 0.003) compared to the control group.

**Table 1:** Clinical characteristics of HFrEF patients and control subjects

Characteristic	Controls *n* = 8	HFrEF *n* = 8	*p-value*
Age (years)	55 ± 1.8	53 ± 4.5	0.939
Gender (% male)	62.5 %	62.5 %	1.000
Weight (Kg)	83.1 ± 6.4	74.6 ± 6.8	0.367
BMI (Kg/m^2^)	27.0 ± 1.3	23.3 ± 1.7	0.088
LVEF (%)	N.A.	31.3 ± 6.0	N.A.
Total cholesterol (mmol/L)	5.02 ± 0.18	4.45 ± 0.51	0.469
HDL (mmol/L)	1.53 ± 0.15	1.25 ± 0.24	0.101
LDL (mmol/L)	3.05 ± 0.29	2.52 ± 0.42	0.363
Triglycerides (mmol/L)	1.00 ± 0.09	1.77 ± 0.17	**0.003**
Glucose (mmol/L)	4.75 ± 0.16	5.51 ± 0.39	0.151
Serum Creatinine (mmol/L)	76.91 ± 3.54	103.43 ± 17.68	0.279
hsCRP (mg/L)	<2.9	22.0 ± 11.3	**0.007**

N, Number of subjects; HFrEF, Heart failure with reduced ejection fraction; BMI, Body mass index; LVEF, Left Ventricular Ejection fraction; HDL, High density lipoprotein; LDL, Low density lipoprotein; hsCRP, high sensitivity C-reactive protein. Significant *p*-values are highlighted in bold. Data are expressed as mean ± SEM

### Morphological analysis of HFrEF myoblasts

**Fig. 1:**
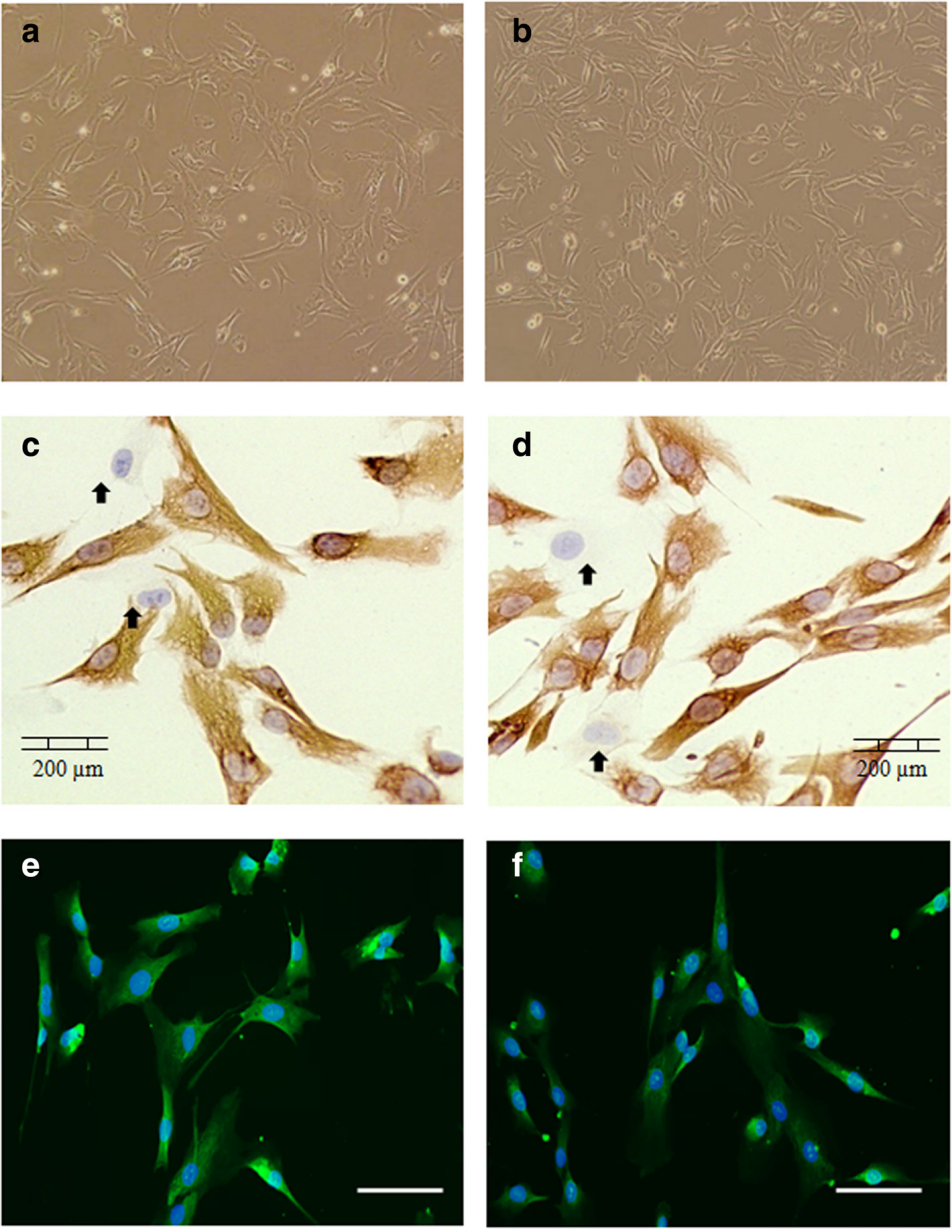
Comparative morphological analysis of HFrEF and control myoblasts. Phase contrast (4X) and immunohistochemical (10X) images of a representative control (**a**, **c**) and HFrEF (**b**, **d**) culture. Desmin-positive myoblasts (*red*) are surrounded by few fibroblasts (*blue, arrows* (⬆)). Actin microfilament organization using phalloïdin (*green*) and DAPI (*blue*) immunostaining of a representative control (**e**) and HFrEF (**f**) culture, 20X magnification. Scale bar = 200 μm. *N* = 6 HF and 6 control cultures

Myoblasts were successfully initiated from six control subjects and six HFrEF patients (Fig. 1a, b). The percentage of desmin-positive myoblasts in each culture was found to exceed 90 % (91.09 ± 1.89 %, HFrEF vs. 92.14 ± 1.76 %, controls; *p* = 0.608; Table 2, Fig. 1a–d). The percentage of desmin-positivity remained similar between groups during the subsequent differentiation step (Table 2). No significant differences in myoblast size or shape were observed between both groups. Myoblasts presented as small elongated cells to flat slightly rounded cells. There were also few large multipolar cells. Cultures were stained for phalloïdin in order to evaluate the actin microfilament arrangement [19]. Stress fibers were also well-organized in myoblasts of HFrEF patients (Fig. 1e, f).

**Table 2:** Myoblast and myotubes characteristics in HFrEF patients versus controls

Characteristic	Controls *n* = 6	HFrEF *n* = 6	*p-value*
Myogenicity (% Desmin positive cells)			
Myoblasts	92.14 ± 1.76	91.09 ± 1.89	0.608
Myotubes	90.80 ± 0.43	92.30 ± 1.19	0.485
Growth characteristics			
Maximum cell index (time in hours)	114.5 ± 12.05	136.5 ± 5.16	0.214
Cell Index (at 90 h)	1.80 ± 0.35	1.0 ± 0.10	**0.032**
Differentiation capacity			
Differentiation Index (DI)	55.49 ± 2.67	46.57 ± 3.29	0.065
Deformed Myotubes Index (DMI)	1.96 ± 0.36	3.10 ± 0.74	0.394
Myogenic Fusion Index (MFI)	57.33 ± 2.40	50.41 ± 1.98	0.093
< 3 Nuclei	20.67 ± 2.45	26.17 ± 2.55	0.266
3–10 Nuclei	56.50 ± 4.59	50.17 ± 5.89	0.574
> 10 Nuclei	21.17 ± 4.64	18.83 ± 2.63	0.905
Viability (% alive cells)			
7-AAD^-^	97.83 ± 0.51	97.05 ± 0.60	0.476
Annexin-V^-^	92.25 ± 1.34	90.59 ± 1.66	0.352

*N* Number of subjects, *HFrEF* Heart failure with reduced ejection fraction. Significant *p*-values are highlighted in bold. Data are expressed as mean ± SEM

### Differentiation of HFrEF myoblasts

**Fig. 2:**
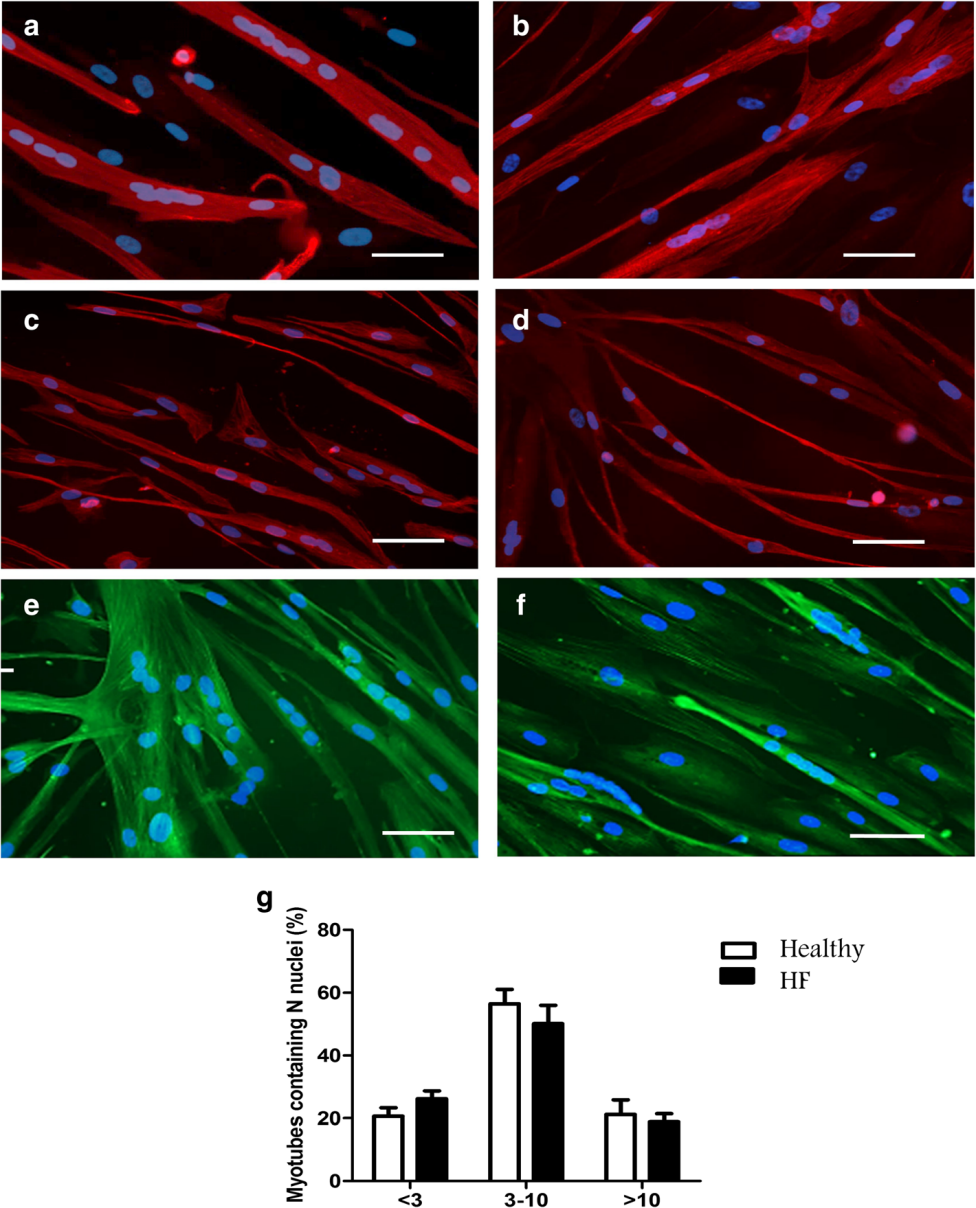
HFrEF and control cultures upon induction of myotubes differentiation. Immunofluorescent images of myogenic cultures from control subjects (*left*) and HFrEF patients (*right*) reacted with antibodies against α-actinin (**a**, **b**), α-tubuline (**c**, **d**) and phalloïdin (**e**, **f**). Magnification of 20X. Nuclei were stained with DAPI (*blue*). Quantification of the number of nuclei (N) present in control and HFrEF myoblast cells (**g**). Scale bar = 200 μm. Data represent mean ± SEM. (*N* = 6 controls and HFrEF patients)

Myotubes differentiation (Fig. 2) started at 48–72 h after the switch from growth to differentiation medium. Multinucleated myotubes became apparent on a background of mononucleated myoblasts. The DI was slightly decreased in HFrEF myoblast cultures compared to controls (46.57 ± 3.29 % versus 55.49 ± 2.67 %), although this observation did not reach significance (*p* = 0.065). Both cultures grew to normal differentiated myotubes marked by a very low percentage of deformed myotubes (DMI of 3.10 ± 0.47 %, HFrEF; 1.96 ± 0.36 %, control, *p* = 0.394). No significant difference in fusion competence was observed between myotubes from HFrEF patients and control subjects (Fig. 2g). Similarly, the size of HFrEF and control myotubes was not statistically different. The maturation and sarcomere assembly were evaluated by means of α-actinin staining and were not different between groups (Fig. 2a). The α-tubulin and actin filament networks were also identical in myotubes of HFrEF patients (Fig. 2d, f) and controls (Fig. 2c, e).

### Quantitative analysis of myoblast viability

The mean numbers of early (Annexin-V^+^/7-AAD^-^) and late apoptotic cells (Annexine-V^+^/7-AAD^+^) were not significantly different between skeletal muscle cells from HFrEF patients and control subjects (*p* = 0.352 and *p* = 0.476, respectively; Table 2).

### Proliferative capacity of HFrEF myoblasts

**Fig. 3:**
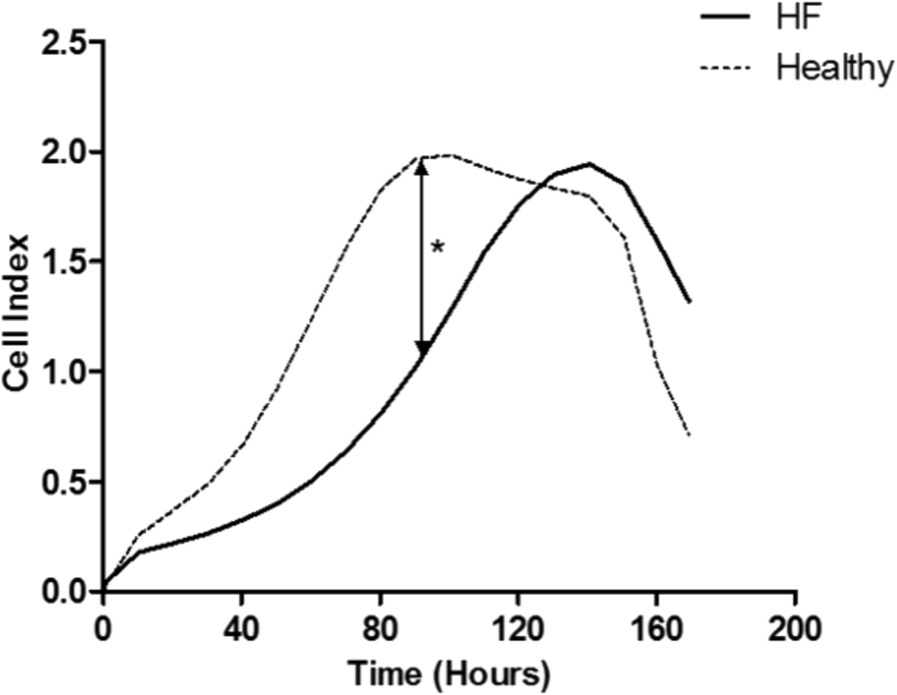
Representative growth kinetics of HFrEF and control myoblasts. Growth curves were generated using the xCELLigence system. (*N* = 8 controls and HFrEF patients) *p < 0.05

The increase in CI from 50 h to 100 h was less pronounced in myoblasts cultures from HFrEF patients. In particular, the mean CI taken during the logarithmic growth phase at 90 h was significantly lower in HFrEF cultures (*p* = 0.032). Myoblasts of HFrEF patients reached their maximum CI at 136.53 ± 5.16 h, whereas myoblasts of control subjects showed a maximum CI at 114.49 ± 12.05 h (*p* = 0.214; Fig. 3).

### Pax3 and Pax7 expression in HFrEF muscle cells

Satellite cell marker Pax3 showed a low expression (1–2 %) in both groups of myotubes cultures, indicating the rare presence of satellite cells (Additional file 1: Figure S1). Pax7 expression at day 0 was present in 72.72 ± 11.80 % of myoblasts from HFrEF patients and in 78.82 ± 8.13 % of myoblasts from controls. The expression level decreased to 51.65 ± 1.86 % and 59.74 ± 2.67 % at day 6 for respectively HFrEF patients and controls (Additional file 1: Figure S1). There was no statistically significant difference in Pax7 expression between groups at either time point.

### Myogenic regulatory factors in HFrEF muscle cells

The expression of the transcription factors MyoD and MRF4 remained stable over time during the process of myogenesis in both myotubes cultures (Additional file 1: Figure S1). A steady increase in myogenin expression within 48 h after the change from proliferation to differentiation medium was detected in both groups, with levels reaching 3-fold of those found on day 0 (Additional file 1: Figure S1). Overall, no significant differences were observed between groups in the percentages of MyoD, Myogenin and MRF4.

### Cellular senescence

**Fig. 4:**
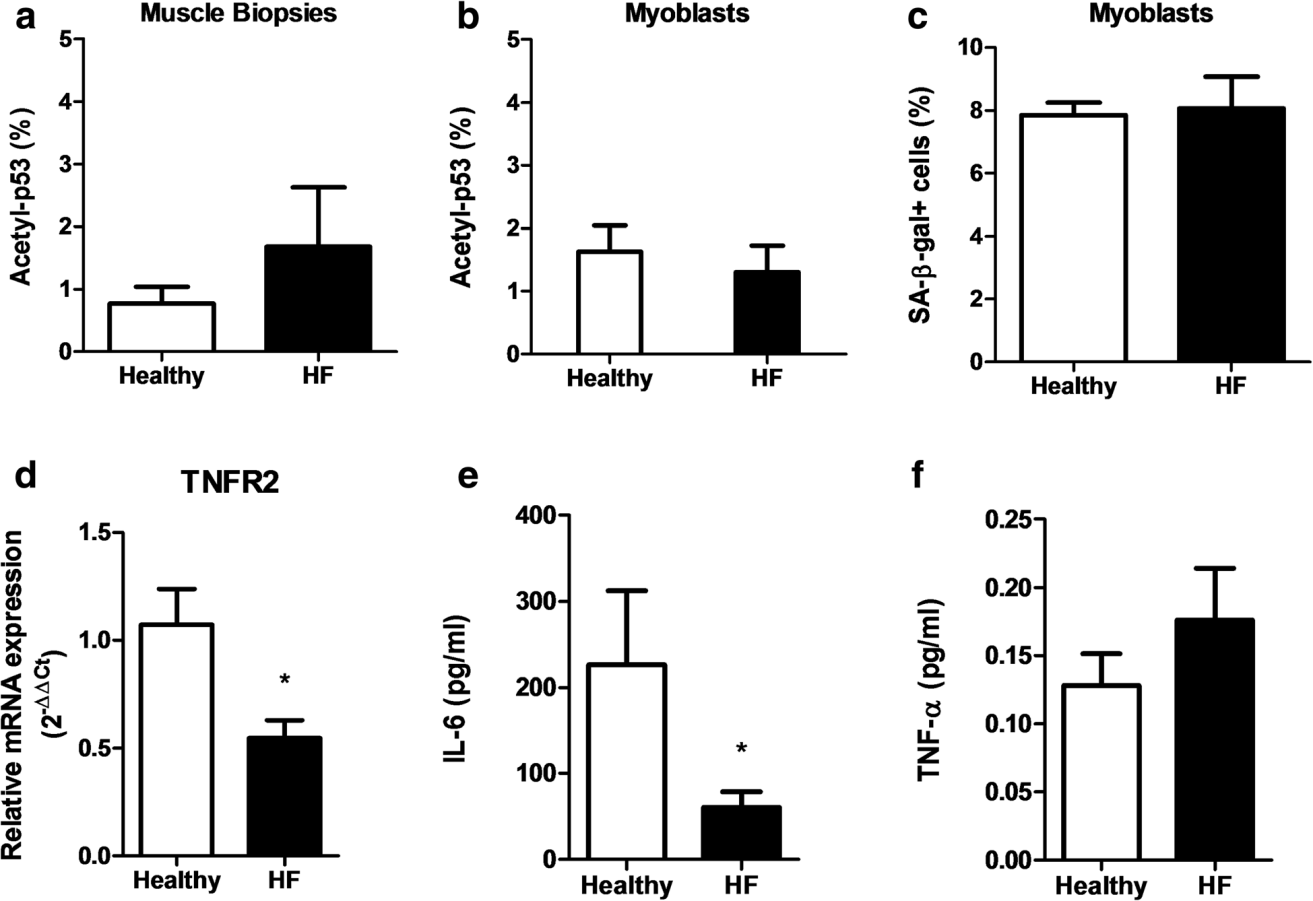
Assessment of senescence and inflammatory characteristics. Representative graphs of mean percentage of acetyl-p53 positive cells in muscle biopsies (**a**) and myoblast cultures (**b**). Quantification of the number of SA-β-gal positive cells present in myoblast cells from control subjects and HFrEF patients (**c**). mRNA expression level of TNFR2 in myoblasts from HFrEF patients and control subjects (**d**). IL-6 (**e**) and TNF-α (**f**) secretion from cultured myoblast cells. Data are mean ± SEM. (*N* = 8 controls and HFrEF patients). *p < 0.05

Muscle biopsies and myoblast cultures from patients and controls displayed no significant differences regarding cellular senescence. First, the expression of acetyl-p53 was similar between groups in both muscle biopsies (1.68 ± 0.95 for HFrEF vs. 0.68 ± 0.25 for controls; *p* = 0.307; Fig. 4a) and myoblast cultures (1.31 ± 0.42 for HFrEF vs. 1.63 ± 0.41 for controls; *p* = 0.590; Fig. 4b). Second, myoblast cultures of HFrEF patients demonstrated an equal amount of SA-β-gal positive cells as controls (*p* = 1.000), which is in accordance with the results for acetyl-p53 (Fig. 4c). Myoblast cultures of HFrEF patients consisted of 8.06 ± 1.01 % SA-β-gal positive cells, whereas cultures of controls demonstrated 7.86 ± 0.39 % SA-β-gal positivity.

### mRNA expression levels of TNFR1 and TNFR2

No statistical difference was detected for the gene expression level of the death receptor TNFR1 between myoblast cultures of patients and controls. In contrast, TNFR2, which functions as a survival receptor, was significantly down-regulated in patient-derived myoblasts (*p* = 0.017; Fig. 4d).

### Cytokine response in cultured myoblasts

Myoblast cultures at 72 h revealed a significantly lower IL-6 secretion (*p* = 0.016) by HFrEF myoblasts (59.95 ± 18.82 pg/ml) compared to control myoblasts (226.11 ± 86.07 pg/ml; Fig. 4e). TNF-α secretion was somewhat increased in HFrEF myoblast cultures, however, this was not statistically significant (Fig. 4f). In addition, no significant difference in IL-10 secretion was observed. Myoblasts did not secrete IL-1β and IFN-γ (mean lower limits of detection of the assays: 0.04 pg/ml and 0.20 pg/ml for IL-1 β and IFN-γ, respectively).

## Discussion

Human skeletal muscle cell cultures have been extensively studied in chronic diseases such as type 2 diabetes, obesity and chronic obstructive pulmonary disease (COPD) [12, 13, 20]. The present study established for the first time primary cell cultures from the skeletal muscle of patients with HFrEF and analyzed these for phenotypic and proliferative characteristics. The main findings can be summarized as follows:

First, primary myoblasts and myotubes from HFrEF patients demonstrate a morphology, myogenic differentiation capacity, viability and senescence that is comparable to muscle cells of healthy donors. Second, myoblasts of HFrEF patients exhibit an altered proliferative and inflammatory activity, supported by the diminished expression of the survival receptor TNFR2 and lower IL-6 secretion.

In the present study we demonstrate that satellite cell-derived myoblasts from HFrEF patients are able to differentiate in vitro into multinucleated myotubes. In addition, we show that skeletal myoblasts and myotubes from patients with HFrEF have a similar size, shape and myogenic differentiation ability as muscle cell cultures from healthy donors. These findings are in line with previous studies in patients with COPD and type-2 diabetes [20, 21]. In these patients, the myotubes’ myogenic fusion index and commitment to terminal differentiation were not different from myotubes of healthy donors. Also, the expression patterns of the myogenic regulatory factors MyoD, Myogenin and MRF4 were similar to healthy donor cultures.

HFrEF myoblasts further demonstrated a delay in proliferation kinetic in comparison to myoblasts of age- and gender-matched healthy donors. Therefore, it could be possible that the satellite cells of the HFrEF patients had already undergone multiple cell divisions in vivo to replenish damaged muscle fibers, resulting in slower culture rates of cell division. A state of cellular senescence, however, was not yet detected in the HFrEF myoblasts. Instead, we observed a reduced expression of the TNF-α receptor TNFR2, but not of TNFR1. Of note, Torre-Amione et al. already reported a diminished expression of myocardial TNFR1 and TNFR2 in patients with advanced HF in 1996 [22]. Recently, patients with HF were found to have elevated levels of circulating soluble TNFR2, which the authors attributed to increased tissue shedding [23]. Furthermore, we observed that the secretion of IL-6 was significantly reduced in comparison to the IL-6 release by the healthy donor myoblasts. TNFR1 and TNFR2 are the two major transducers of TNF-α signals. Ligation of TNF-α with TNFR1 leads to caspase activation and induces apoptotic cell death [24]. In contrast, TNFR2 signaling activates nuclear factor-kappa β (NF-kβ) and signal transducer and activator of transcription 3 (STAT3), and thereby promotes cell proliferation, cytokine production and cell survival [25]. IL-6 is a pleiotropic cytokine that is, among other cells, also produced by growing myofibers and associated satellite cells. It stimulates the robust activation and proliferation of satellite and myoblasts cells in both autocrine and paracrine manners via STAT3 signaling, and therefore acts as a novel mediator in controlling muscle regeneration [26–28]. In this regard, Serrano et al showed that if IL-6 is genetically deleted, satellite cell proliferation and migration become attenuated, leading to a reduction in myofiber size [29]. Therefore, based on our findings and the results from previous studies, it seems that persistent exposure of skeletal muscle cells to high systemic and local levels of TNF-α in HFrEF induces a refractory cell state, provoking a decrease in muscle cell secretion of IL-6 and a reduction in TNFR2 expression, thereby attenuating cell proliferation, and this plausibly without affecting the endogenous synthesis of IL-6 [30, 31]. In this regard, in vitro studies by Hamilton et al. demonstrated that a single 10-h incubation step of colon cancer cells with TNF-α leads to endogenous IL-6 through NF-kβ activation and promotes TNFR2 expression via the autocrine effects of IL-6 [32]. Yet, repeated exposure of cells to TNF-α was shown to weaken the secretion of IL-6 [33]. Hence, the lower secretion of IL-6 may well contribute to the decrease in proliferative competitiveness of the HFrEF myoblasts.

Another potential mechanism underlying reduced myoblast proliferation in HFrEF is the lack of physical activity in these patients [34–37]. It has been demonstrated that both physical training and a single bout of exercise positively influence satellite cell function, myoblast proliferation, and cytokine expression and secretion [38–40]. Resistance training, for instance, increases satellite cell content and activation status, and stimulates satellite cell proliferation by IL-6 induced activation of STAT3 signaling [41, 42]. Recently, Begue et al. also indicated that resistance exercise training promotes satellite cell proliferation by IL-6 induced activation of STAT3 signaling [43]. Furthermore, metabolic disturbances such as hyperglycaemia, insulin resistance, mitochondrial dysfunction and a decreased activation of 5′ adenosine monophosphate-activated protein kinase (AMPK) and p38 mitogen-activated protein kinase (MAPK) were shown to impair the proliferation of porcine myoblasts and C_2_C_12_ mouse myoblasts [44–49]. In addition, growth differentiation factor myostatin was shown to negatively regulate the self-renewal of satellite cells and to inhibit C_2_C_12_ muscle cell activation, proliferation, myogenic differentiation and protein synthesis [50–52]. As such, distinct mechanisms might be involved in the altered inflammatory and proliferative actions of HFrEF myoblasts.

### Study limitations

Cells from both HFrEF patients and control subjects were cultured under the same in vitro environmental conditions which did not reflect the prevailing inflammatory milieu to which skeletal muscles of HFrEF patients are exposed to in vivo. Circulating TNF-α was not measured, however, previous studies have indicated increased serum TNF-α levels in HFrEF [1, 22, 23]. HFrEF patients were on optimal medical treatment and thus, were administered a number of pharmacological agents including ACE-inhibitors, beta-blockers, diuretics and statins. In this regard, and although well-tolerated by the majority of patients, evidence has indicated that statins may affect (either positively or negatively) skeletal muscle function [53–56]. In our study, we observed no differences in myoblast number, morphology, or differentiation capacity among HFrEF patients on statin therapy (75 % of patients) and those not taking the drug (25 %). In addition, it has been shown that chronic kidney disease (CKD), diabetes mellitus and COPD, common co-morbidities in patients with HFrEF, may affect muscle cell parameters including proliferation and differentiation capacities [20, 57, 58]. Therefore, HFrEF patients with major comorbidities were excluded from the study. Finally, results are to be confirmed in a wider range of patients with HFrEF.

## Conclusion

In this study, we show that myoblasts derived from HFrEF patients have altered proliferative and reduced anti-inflammatory activity if compared to healthy donor cells. We believe that the in vitro cultivation of biopsy-derived primary skeletal muscle myoblasts and myotubes is a promising tool for future research on muscle wasting in HFrEF.

## Abbreviations

AAD, Aminoactinomycin; ACE, Angiotensin fPIconverting enzyme; AMPK, 5′ adenosine monophosphate-activated protein kinase; B2M, Beta-2-microglobulin; BMI, Body mass index; CI, Cell index; CKD, Chronic kidney disease; COPD, Chronic obstructive pulmonary disease; DAPI, 4′,6-diamidino-2-phenylindole; DI, Differentiation index; DMEM, Dulbecco’s modified eagle medium; DMI, Deformed myotubes index; ELISA, Enzyme-linked immunosorbent assay; FITC, Fluorescein isothiocyanate; HDL, High-density lipoprotein; HF, Heart failure; HFrEF, Heart failure with reduced ejection fraction; hsCRP, High sensitivity C-reactive protein; IFN-γ, Interferon-γ; IL, Interleukin; LDL, Low-density lipoprotein; LVEF, Left ventricular ejection fraction; MAPK, Mitogen-activated protein kinase; MFI, Myogenic fusion index; MSD, Meso Scale Discovery; NF-kβ, Nuclear factor-kappa β; NYHA, New York Heart Association; RT, Room temperature; RTCA, Real-Time Cellular Analysis; RT-PCR, Reverse transcriptase-polymerase chain reaction; SA-β-gal, Senescence-associated beta-galactosidase; SEM, Standard error of the mean; SKGM, Skeletal muscle growth medium; SPSS, Statistical package for the social sciences; STAT3, Signal transducer and activator of transcription 3; TBP, TATA box binding protein; TNF-α, Tumor necrosis factor-α; X-Gal, 5-bromo-4-chloro-3-indolyl *β*-D-galactopyranoside.

## Additional file

Additional file 1: Figure S1. Differentiation of HFrEF myotubes. Representative graphs showing the expression pattern of Pax3 (A), Pax7 (B) and the myogenic regulatory factors MyoD (C), Myogenin (D) and MRF4 (E) in muscle cells cultivated from HFrEF patients and control subjects following 6 days in differentiation medium. Satellite cell marker Pax3 (A) showed a low expression in both groups of myoblasts cultures indicating the relatively absence of satellite cells. A slightly increased Pax7 expression was observed within 48 h after having changed the proliferation medium to differentiation medium (B). As the myoblasts cultures matured, the level of this transcription factor gradually decreased whereby the reduction in Pax7 expression was more pronounced in HFrEF, although not statistically significant myoblasts. Expression of transcription factors MyoD and MRF4 remained stable over time during myogenesis (C, E). A steady increase in myogenin expression within 48 h after the change from proliferation to differentiation medium was detected in both groups with levels reaching 3-fold of those found on day 0 (D). Data are mean ± SEM. (*N* = 6). (PDF 104 kb)
