# Supplementary material for: Primary skeletal muscle myoblasts from chronic heart failure patients exhibit loss of anti-inflammatory and proliferative activity
Source: BMC Cardiovasc Disord. 2016 May 26;16:107. doi: 10.1186/s12872-016-0278-3 (PMC4880810; doi:10.1186/s12872-016-0278-3)
Supplement: Additional file 1: Figure S1. — Differentiation of HFrEF myotubes. Representative graphs showing the expression pattern of Pax3 (A), Pax7 (B) and the myogenic regulatory factors MyoD (C), Myogenin (D) and MRF4 (E) in muscle cells cultivated from HFrEF patients and control subjects following 6 days in differentiation medium. Satellite cell marker Pax3 (A) showed a low expression in both groups of myoblasts cultures indicating the relatively absence of satellite cells. A slightly increased Pax7 expression was observed within 48 h after having changed the proliferation medium to differentiation medium (B). As the myoblasts cultures matured, the level of this transcription factor gradually decreased whereby the reduction in Pax7 expression was more pronounced in HFrEF, although not statistically significant myoblasts. Expression of transcription factors MyoD and MRF4 remained stable over time during myogenesis (C, E). A steady increase in myogenin expression within 48 h after the change from proliferation to differentiation medium was detected in both groups with levels reaching 3-fold of those found on day 0 (D). Data are mean ± SEM. (N = 6). (PDF 104 kb) [file 12872_2016_278_MOESM1_ESM.pdf]

SUPPLEMENTAL MATERIAL

Figure S1

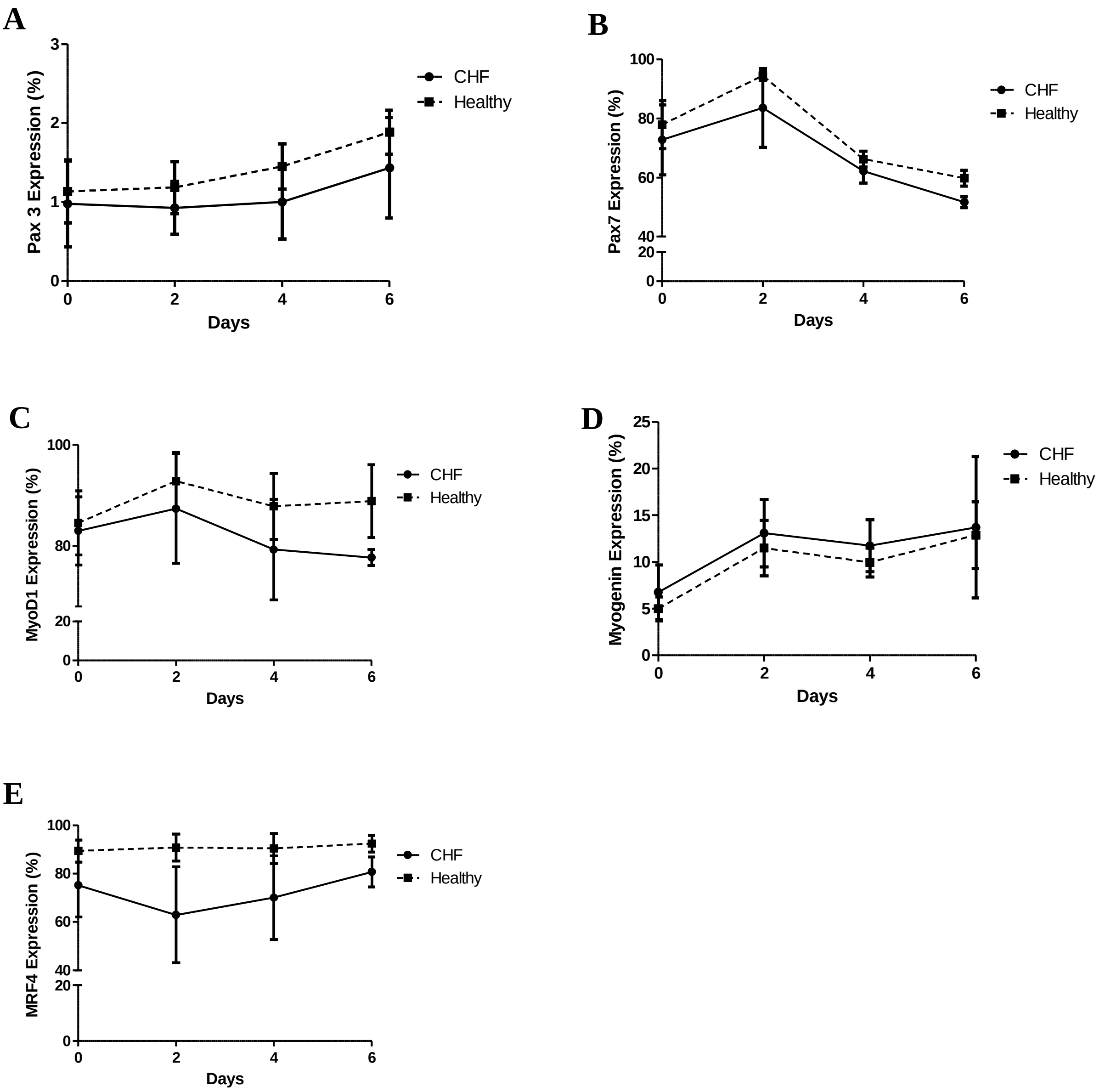

Figure S1. Representative graphs showing the expression pattern of Pax3 **(A)**, Pax7 **(B)** and myogenic regulatory factors MyoD **(C)**, Myogenin **(D)** and MRF4 **(E)** in muscle cells obtained from CHF patients and control subjects following six days in differentiation medium. Satellite cell marker Pax3 **(A)** showed a low expression in both myoblasts cultures, indicating the relatively absence of satellite cells. A slightly increased Pax7 expression was observed within 48h after having changed the proliferation medium to

differentiation medium **(B)**. As the cultures matured, the level of the Pax7 transcription factor gradually decreased whereby the reduction in Pax7 expression was more pronounced in CHF myoblasts. Expression of transcription factors MyoD and MRF4 remained stable over time during myogenesis **(C, E)**. A steady increase in myogenin expression within 48 h after the change from proliferation to differentiation medium was detected in both groups with levels reaching 3-fold of those found on day 0 **(D)**. Data are mean ± SEM. (N=6)
